# webGQT: A Shiny Server for Genotype Query Tools for Model-Based Variant Filtering

**DOI:** 10.3389/fgene.2020.00152

**Published:** 2020-03-03

**Authors:** Meharji Arumilli, Ryan M. Layer, Marjo K. Hytönen, Hannes Lohi

**Affiliations:** ^1^Department of Veterinary Biosciences, Department of Medical and Clinical Genetics, University of Helsinki, Helsinki, Finland; ^2^Genetics Research Program, The Folkhälsan Research Center, Helsinki, Finland; ^3^Department of Computer Science, University of Colorado, Boulder, CO, United States; ^4^The BioFrontiers Institute, University of Colorado, Boulder, CO, United States

**Keywords:** variant, filtering, R package, shiny server, GQT, webGQT, Bigdata

## Abstract

**Summary:**

Genotype Query Tools (GQT) were developed to discover disease-causing variations from billions of genotypes and millions of genomes, processes data at substantially higher speed over other existing methods. While GQT has been available to a wide audience as command-line software, the difficulty of constructing queries among non-IT or non-bioinformatics researchers has limited its applicability. To overcome this limitation, we developed webGQT, an easy-to-use tool with a graphical user interface. With pre-built queries across three modules, webGQT allows for pedigree analysis, case-control studies, and population frequency studies. As a package, webGQT allows researchers with less or no applied bioinformatics/IT experience to mine potential disease-causing variants from billions.

**Results:**

webGQT offers a flexible and easy-to-use interface for model-based candidate variant filtering for Mendelian diseases from thousands to millions of genomes at a reduced computation time. Additionally, webGQT provides adjustable parameters to reduce false positives and rescue missing genotypes across all modules. Using a case study, we demonstrate the applicability of webGQT to query non-human genomes. In addition, we demonstrate the scalability of webGQT on large data sets by implementing complex population-specific queries on the 1000 Genomes Project Phase 3 data set, which includes 8.4 billion variants from 2504 individuals across 26 different populations. Furthermore, webGQT supports filtering single-nucleotide variants, short insertions/deletions, copy number or any other variant genotypes supported by the VCF specification. Our results show that webGQT can be used as an online web service, or deployed on personal computers or local servers within research groups.

**Availability:**

webGQT is made available to the users in three forms: 1) as a webserver available at https://vm1138.kaj.pouta.csc.fi/webgqt/, 2) as an R package to install on personal computers, and 3) as part of the same R package to configure on the user's own servers. The application is available for installation at https://github.com/arumds/webgqt.

## Introduction

Exome sequencing, genome sequencing, and gene panel sequencing methods have become the de facto methods for studying the heritability of human and non-human genetic diseases involving small pedigrees to large-scale population cohorts. The current wealth of sequenced genomes produces billions of genotypes in the variant call format (VCF), requiring publicly available programs to effectively and rapidly filter candidate variants for personalized disease and population genomics. There are tools to analyze large VCF files, such as GEMINI (Paila et al., 2013), *canvasDB* (Ameur et al., 2014), VCF-miner (Hart et al., 2016) and VarSifter (Teer et al., 2012), which have specific pros and cons in terms of large file handling, query time, graphical user interface (GUI), memory requirement, and application to non-human genomes when compared with each other. A recent study has shown that VCF-Explorer (Akgun and Demirci, 2017), a GUI program, has out-performed the standard queries in speed. On the other hand, Genotype Query Tools (GQT), a command line software (Layer et al., 2016), was developed to query and scale-up to the billions of loci from the UK 100,000 Genomes Project (Samuel and Farsides, 2017) and the expected millions of microbial, plant and animal genomes (Stephens et al., 2015) using a Word-Aligned Hybrid (WAH) compressed bitmap index. The GQT algorithm uses a sample-centric indexing strategy that is orders of magnitude faster than existing methods to query genotypes, phenotypes, and familial relationships. The 1000 Genome Project Consortium has extended to 2,504 individual genomes in Phase 3 and GQT has become an integral tool in the 1000 Genomes Project to expedite such massive data sets (Genomes Project et al., 2015). Additionally, users use the GQT command line interface to filter inherited variants among small pedigrees, case-control variant filtering, and comparing variants among different cohorts. While GQT is available to a wide audience as command line software, the difficulty in constructing queries faced by non-IT or non-bioinformatics researchers has limited its applicability among many users.

Many re-sequencing projects complemented with publicly available variant data sets are on the scale of several thousands of genomes. Tasks such as variant calling, data processing and management, and storing the genome data are most commonly implemented by experienced bioinformaticians within the research groups. The billions of variants from such large-scale projects offer a challenge in extracting candidate variants from specific families or groups specific to a research project. Furthermore, the presence of additional samples or controls within the cohort is an invaluable resource that allows users to filter out the common variants. Specifically, for non-human data sets there are no or few population frequency databases like 1000G Genomes Project (Genomes Project et al., 2015), ExAC (Lek et al., 2016), and gnomAD (https://doi.org/10.1101/531210) to greatly reduce the number of potential causal variants for further downstream analysis. However, the extraction of candidate variants from the entire variant database to a manageable subset still requires the assistance of bioinformaticians in every project associated with the data set. For example, consider a research group with “n” geneticists having more than one family to study from the samples within the data set. And each researcher requiring to analyze or filter variants in “x” different ways per family. This requires the bioinformatician to perform “n*x“ filtering tasks per family which is daunting and repetitive. A central installation of webGQT on institutional servers deployed with user data sets would enable geneticists, clinicians or researchers with life science backgrounds to filter for candidate disease variants without requiring computational skills or installing R or webGQT.

Here, a web server for GQT (webGQT) is developed to support inheritance model-based filtering among a family or a group of individuals in the study cohort utilizing the GQT index files to reduce computation time. Specifically, webGQT is developed with an emphasis to provide a web server that can be utilized as a common platform across members of a research group without requiring independent access to the large variant databases. We present a graphical user-interface (GUI) application to identify candidate disease-causing variants according to user-defined inheritance patterns that include dominant, recessive, *de novo* inheritance models, population comparisons, and sample-based filtering from large cohorts. The pedigree-based studies and case-control studies aim to identify rare disease-causing variants that are present in affected individuals and absent in un-affected individuals. The population-based studies filter for variants with differential MAFs among two populations. We present several use-cases available with webGQT, representing Mendelian inheritance models, case-control studies, and population studies that can be implemented on human and other model species. This is validated by using webGQT on the data from the study (Salmela et al., 2018), which identified the causal variant segregating in a recessive inheritance pattern. The key features include:

1. scale-up to efficiently query the stored genotypes in GQT index files from the upcoming millions of genomes (Stephens et al., 2015; Samuel and Farsides, 2017)
2. rapid filtering of massive data sets relative to applications such as *canvasDB* (Ameur et al., 2014)
3. Mendelian inheritance disease models for pedigree-based studies, case-control studies, and complex population comparisons
4. control for minor allele-frequencies (MAF) within control cohorts
5. cross-species support (unlike few other tools that are limited to only human data sets) (Paila et al., 2013) and
6. the flexibility of running as either a web service or a standalone webserver for use within and between research groups.

## Methods

### Overview of webGQT

**Figure 1:**
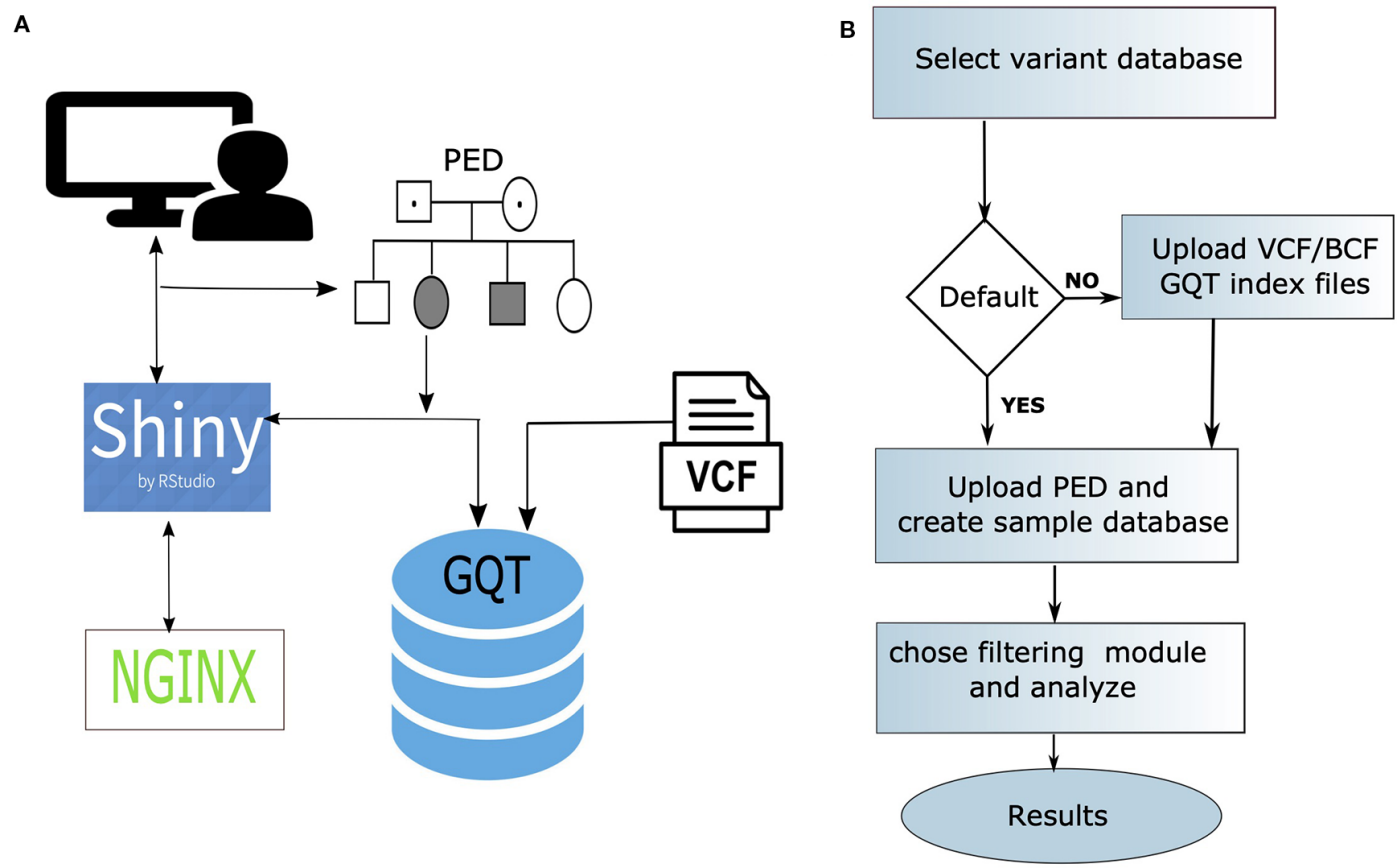
**(A)** An overview of the architecture of the webGQT system. The variant information is stored in as GQT index files. The user performs the query on the GQT index files from the GUI provided by the shiny server and the results are returned to the user via GUI. The whole application is secured with a Nginx front-end proxy server to serve https requests. **(B)** The three-step workflow of implementing webGQT is shown here: 1) selecting the default data set (e.g., 1000 Genomes) or uploading GQT indexed files, 2) uploading phenotype file (PED) and creating sample database, and 3) choosing a module and performing variant filtering.

**Figure 2:**
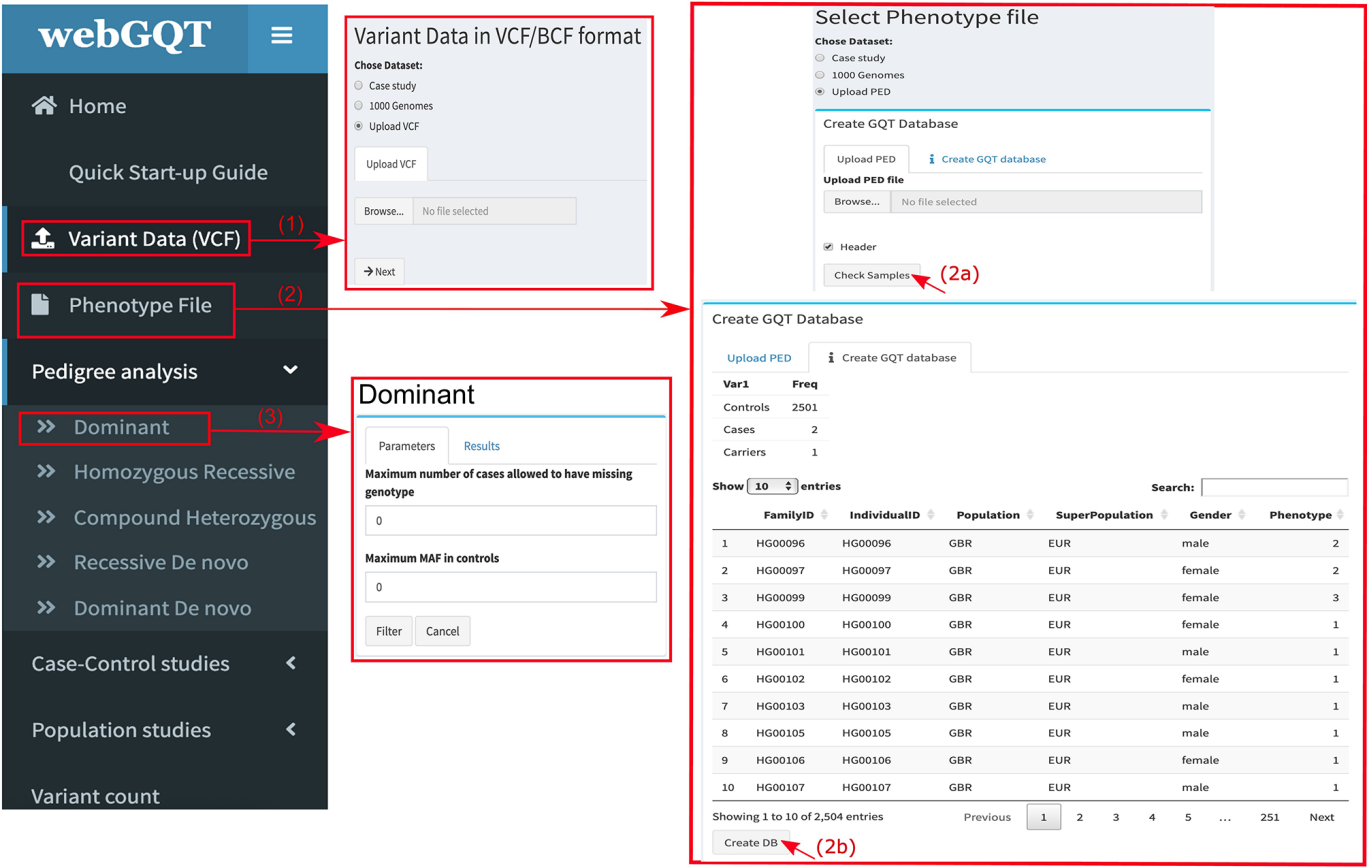
Figure showing the work flow of webGQT via user interface: (1) interface showing the user upload panel for input variant data. The user can choose the default data set or upload GQT indexed files. (2a) Interface showing the user upload panel of the phenotype file. The user uploads the PED file by clicking “Browse” button. (2b) After uploading, the phenotype file is rendered as data table with the sample selection information. The user is then required to create a phenotype sample database by clicking “CreateDB” and (3) The user choses a filtering module and applies the available parameters of the corresponding module and finally filters the variants. A dominant analysis module filter is shown in the figure.

webGQT utilizes GQT indexed files as the variant database and serves as a webtool to filter candidate variants more rapidly by the user criteria. webGQT is implemented using the R Shiny server, which provides a graphical user interface (GUI), and variant database queries are performed using GQT in the backend, with the filtered variants rendered as data tables in the GUI. A new installation of webGQT can be done on a personal computer using Rstudio as an R package. Alternatively, the users can host their own version of webGQT on a local or remote server with nginx HTTP server to act as front-end proxy to shiny server. Additionally, we host the application on our institutional server where users can explore the features and upload the data for analysis. Each data upload creates a new instance and is only viewable to the specific user during a single browser session. In order to allow multiple users to access the application, the data upload size per user is limited to 50GB. An overview of the webGQT system architecture is shown in **Figure 1A**. The webGQT application has been extensively tested on Chrome, Firefox, and Safari browser environments. The workflow for implementing webGQT is shown in **Figures 1B** and **2**. In the first step, the user selects the default data set or uploads the variant database. Subsequently, a PED file with sample relationships or affection status is uploaded, and a sample database is created. In the final step, a filtering module is chosen and analyzed to obtain the variant results (**Figure 1B**). The GUI provides documentation for each module about data preparation, input file format specifications and parameters to be used in each module. The GUI of webGQT is shown in **Figure 2**, illustrating the workflow assuming a dominant inheritance mode of filtering.

### Input Specifications

The Variant Call Format (VCF) has been the standard for storing genome-variation from variant calling algorithms like GATK and SAMtools. webGQT allows users to filter single nucleotide variants (SNVs), short insertions/deletions (INDELs) and also copy number variants and structural variations (SV) generated in the standard VCF format. The user can generate a merged multi-sample VCF file from cohorts that have been called using different variant callers. For example, bcftools {http://github.com/samtools/bcftools} can be used to merge SNV and INDEL VCF files from disparate SNV variant callers and Parliament2 {https://doi.org/10.1101/424267} can be used to combine variant calls from multiple SV callers to generate an accurate VCF file for indexing with GQT. Furthermore, the user can also merge SNV and SVs into standard multi-sample VCF format files for indexing and querying.

In a VCF, variants are represented in a “variant-centric” manner where each row corresponds to a variant and the genotypes of individuals at that locus. The “variant-centric” analyses allow the retrieval of specific variants based on chromosomal location. In this “variant-centric” format, comparing the genotypes of two individuals is highly inefficient. The comparison must load every row (variant) but only accessing two elements per row. To improve our ability to query by genotypes and other individual-level attributes (phenotypes, affection status, population groups), we transpose variants so that each row corresponds to an individual and their genotypes as columns for all loci. In this “sample-centric” format, comparing two individuals requires accessing only two rows and all values in those rows. The transposed data is then sorted by allele frequency to improve compression of the data. Finally, Word-Aligned Hybrid (WAH) compression strategy enhances query performance without inflation.

GQT indexes the standard VCF file with the command “gqt convert bcf -i input.vcf/input.bcf” to allow “sample-centric” variant filtering which is used as input by webGQT. GQT indexing creates three metadata files; a compressed index (.gqt), a compressed summary of the variant metadata (.bim), and a variant ID file (.vid) file, which are only a fraction of the source VCF/BCF, and thus saves storage space and improves performance query over the existing methods. The conversion pre-process efficiently indexes and compresses the variant data in VCF/BCF format as described in the original study (Layer et al., 2016). Creating GQT index files is a one-time pre-processing step on the target VCF/BCF file upon which numerous queries can be performed by multiple users from different projects.

Second, webGQT requires a PED file that defines the sample information, phenotypes, population groups, case-control status, and relationships of the samples in the target VCF/BCF file. The PED file should contain the tab-separated fields of IndividualID, Phenotype, Sex, and Population in the first line to perform pedigree analysis, case-control studies, and population comparisons (**Supplementary Table 1**). The IndividualID and Phenotype fields are mandatory for analysis in pedigree and case-control modules. The “IndividualID” column represents the sample names in the target VCF/BCF file. The “Phenotype” column represents the affection status of the samples. The phenotype coding for the samples in the PED file is as follows: 0 represents samples to exclude from the analysis; 1 represents unaffected parent or unaffected offspring/siblings or other unaffected individuals (controls); 2 represents affected offspring (cases); and 3 represents affected or carrier parents. IndividualID, Population, and Sex fields are mandatory for population modules while other optional fields can be present. Sex is coded as 1 for males, 2 for females, and NA if unavailable. When PED files are uploaded, the interface allows users to review the sample relations and affection statuses from the data table before creating the sample database. A sample database has to be created to enable rapid variant filtering of cohorts with different sample attributes in the associated VCF/BCF file. The sample database created in the PED file complements the GQT index files created in the original VCF or binary VCF (BCF) file to identify the compressed target sample bitmaps and retrieve the genotype bitmaps in VCF format.

### webGQT Analysis Modules

Establishing the causality of the variant as disease-causing variant requires hypothesizing analysis strategies with different modes of inheritance across pedigree, case-control, and population modules in the GUI. Each module is constructed with independent analysis models for studying with familial cases or trios or non-familial case-control samples or group level comparisons.

The models of pedigree module:

- **Recessive model:** In autosomal recessive disorders, the affected individual inherits one pathogenic mutation from each parent (the parents are carriers). webGQT filters for such variants, where the parents are heterozygous carriers (Phenotype=3) for the variant and the affected individual (Phenotype=2) has a homozygous state.
- **Compound heterozygous**: In the case of such a recessive model, we implemented a compound heterozygous variant filter on a single variant level. To be able to perform this analysis, the input VCF requires the genotypes of all individuals in the trio (off-spring and parents). This model filters for at least two heterozygous variants in the affected offspring (Phenotype=2) for which one of the parents is heterozygous and the other parent should have a wild-type genotype (both parents, Phenotype=3). The control cohorts can be further used to filter the common variants in the general population (Phenotype =1) using the MAF cut-off parameter.
- **Dominant model:** In autosomal dominant disorders, the affected individual inherits a single variant from the affected parent. webGQT filters for such variants, where the affected parent (Phenotype=3) and the affected child/sample (Phenotype=2) are heterozygous. Ideally, the affected parent genome is required to accurately retrieve potential dominantly inherited variants. In the absence of the affected parent, the user can switch to the case-specific module to filter for the heterozygous variants specific to the affected individual while the unaffected parent (Phenotype=1) or other unaffected individuals (Phenotype=1) are used as controls.
- **Recessive *de novo*:** This module identifies the mutations that are absent in the parents (Phenotype=3) and are novel homozygous mutations in the offspring (Phenotype=2). Common variants in the general population can be filtered out using the unaffected control samples ((Phenotype=1) and the MAF parameter.
- **Dominant *de novo*:** This module identifies the mutations that are absent in the parents (Phenotype=3) and occur as novel heterozygous mutations in the offspring (Phenotype=2).

Ideally, the analysis models in the pedigree module would require the genotypes from both the parents to infer recessively inherited variants. However, in the absence of one parent's genotype, webGQT can still mark a possible inherited variant based on only one parent's genotype. Quite often, sequencing errors or low-quality regions produce missing or unknown genotypes that lead to the loss of information in candidate regions in the affected samples. To rescue variants in these regions, the user can choose to allow missing genotypes in the affected individuals. Further, the availability of unaffected samples (Phenotype=1) or control cohorts (i.e., 1000 Genomes and gnomAD) (Genomes Project et al., 2015; Lek et al., 2016) can allow the user to filter out common variants using a minor allele frequency (MAF) cutoff. Specifically, while studying the isolated populations, the MAF parameter allows to adjust the carrier frequency in the control group. The missing genotypes and MAF parameters are available in all filtering modules and are important contributors to webGQT.

The models in case-control module:

- **Case-specific:** This model filters the variants that are present only in the affected individuals (Phenotype=2) and absent in the unaffected individuals (Phenotype=1). The user can specify the genotype state of the variants from HOM_ALT, HET, HET HOM_ALT to retrieve case-specific homozygous variants, heterozygous variants and all non-reference variants, respectively. The user can also control for missing genotypes in this model.
- **Cases shared:** This model filters the variants that are shared in the cases (affected individuals, Phenotype=2). By default, this module does not consider variants in the unaffected individuals (Phenotype=1). However, users can set a maximum MAF cutoff in the control cohort to retrieve variants shared by the cases and some controls. This model represents a relaxed version of the case-specific module, allowing users to control for genotypes (HOM_ALT, HET, HET HOM_ALT, UNKNOWN) and MAF.

For the population module, the PED file should have the population groups and sex specified with the fields Population and Sex, respectively. This module filters the variants between two populations defined in the PED file by allele frequencies or individual count or by sample.

- **Allele-frequency:** The user can filter variants that are present in the Finnish (FIN) male population occurring at minimum MAF threshold and occurring in the British (GBR) male population at a maximum MAF cutoff.
- **Individual count:** The user can filter the variants by genotype (HET or HOM_ALT) that are present in minimum percentage of individuals in the Finnish population and occurring in British population in at most percentage of individuals.
- **Sample:** This model can be used to retrieve variants present in specific samples. The sample names can be selected from the dynamically populated dropdown list from the IndividualID field defined in the PED file. The user can ask to retrieve specific genotypes (HOM_ALT, HET, HET HOM_ALT) among the selected samples. Further, a minimum number of samples with the chosen genotype can be set using the “Count” parameter.

**Variant count**: The variant count module allows users to count the variants of selected genotypes (HOM_ALT, HET, HET HOM_ALT) in a specific sample selected from the dropdown list. This operation is orders of magnitude faster compared to the other modules as it only gives the count of the variants instead of writing the output to VCF file.

### Data From 1000 Genomes Project

We provide implementation of all the available modules using webGQT with data from 1000 Genomes Phase 3 release. This release includes 8.4 billion variants from 2,504 individuals across 26 populations (Genomes Project et al., 2015). To load 1000 genome phase 3 variant data into webGQT, the VCF files have been converted to bcf format, indexed, and compressed using GQT command “gqt convert bcf -i ALL.wgs.phase3_shapeit2_mvncall_integrated_v5.20130502.genotypes.bcf” which creates three metadata files with the file extensions “.gqt,” “.bim,” and “.vid.” The indexing and compressing is a one-time pre-processing step on the data set. Subsequently, a PED input file describing the samples in the VCF is used to create the sample database with the GQT command “gqt convert ped -i ALL.wgs.phase3_shapeit2_mvncall_integrated_v5.20130502.genotypes.bcf -p 1kg.phase3.ped.” The index files are then used to perform multiple queries on the variant data by using PED files defining the affection status of the samples from different projects. For the demo, we have randomly defined HG00096 and HG00097 samples as affected offspring (Phenotype=2), HG00099 sample as affected parent (Phenotype=3) and the remaining 2,501 individuals as unaffected controls (Phenotype=1) and created the sample database. The GQT index files for Phase 3 of the 1000 Genomes project are available for download from the homepage of the web server.

### Hardware and Software

webGQT is implemented using R Shiny server to provide a graphical user interface to query variants stored in GQT index files. GQT utilizes SQLite database to query the samples and phenotypes with the GQT indexed variant files. The webGQT installations and analyses were performed on an Ubuntu 18.04.1 64-bit system, with 16 CPUs and 80 Gb RAM with 700 Gb ephemeral hard disk and GQT version 5.5.29 and R version 3.4.4 (https://vm1138.kaj.pouta.csc.fi/webgqt/). webGQT can be installed as an R package on Linux/Mac OS operating computers as well as on local servers to serve within a group of users. The web application is available for download and installation as an R package with instructions at https://github.com/mehararumilli/webgqt.

## Results

### Select Variant Database

The first step in using webGQT is to select the type of variant database to perform the query. webGQT is deployed with 1000 Genomes phase 3 variant data set and the user can query the phase 3 data set by choosing “1000 Genomes” as the input data set. This provides a platform to query 2,504 individuals from 26 populations, simply by choosing the desired samples in subsequent steps for a specific query. To use webGQT on a custom data set through the web server, the user is either required to upload the GQT index files by clicking the “Upload VCF” button or to deploy the application with a default data set on their personal computer or server. The latter functionality allows the provision of web services within research groups and helps clinicians to explore patient data through their local installation on their own servers or computers. The “Upload VCF” page also shows the instructions to prepare the input GQT index files on the target VCF/BCF.

### Creating and Querying by Sample Database

The phenotype file page is used to input the PED file to query the GQT indexed variant files. An SQLite sample database of the PED file is created. The sample database has a single table with sample name and sample position. The sample database created on the pedigree file (PED) complements GQT for rapid and complex queries based on the individual's phenotype/ancestries/relationships, provided in the phenotype file. By clicking on the “Browse” button the user uploads a PED file (.ped extension) and can visualize the PED file as a data table by clicking “View samples” (**Supplementary Figure 1A**). This gives a summary of the number of cases, controls or carriers used in the study and the “Phenotype” column in the data table can be used to review the affection status of the individuals (**Supplementary Figure 1B**). Subsequently, by clicking the “ÇreateDB” button, a sample database that describes the samples in the target BCF file is created. Once the sample database is created, the subset of variants that meet the user criteria can be quickly identified by choosing one of the analysis modules. An example of performing variant filtering with a dominant module is shown in **Supplementary Figure 2A** with the progress bar displayed at the bottom of the page.

### Output

When webGQT successfully completes the query, the results page is displayed, which gives: 1) Summary: a count of the total variants and a summary table of variants annotated across different functional regions (if annotated) (**Supplementary Figure 2B**); 2) Table: a data table which displays the filtered variants in a navigation page that displays the selected number of entries per page (**Supplementary Figure 2C**); and 3) a bar plot summarizing the total retained variants by functional region when the variants are annotated (**Supplementary Figure 2D**). webGQT currently supports the annotation terms from ANNOVAR and snpEff to summarize the functional annotations. The gene summary table and the bar plot are empty if the gene annotations are absent in the input VCF.

In the results “Table” panel, the user can apply sorting of individual variant columns to inspect the results or can download the results from the download buttons (**Supplementary Figure 2C**). Download VCF button retrieves the output in the standard VCF (.vcf) format, which can be used with any third-party tools compatible with VCF input, e.g. bcftools for custom filtering, VEP for gene annotation. “Download Table” generates a text format (.xls) file of variants which has a similar format to the variants displayed in the table pane in the results page. This file can be analyzed in Excel to further fine-tune the results.

### Case-Study: Detection of a Disease-Causing Mutation in Model Species

We have implemented webGQT on the variant data from the recent study “A novel *KRT71* variant in curly‐coated dogs” (Salmela et al., 2018), which illustrates the usefulness of the tool that identifies the causative variant for curly coat type in specific dog breeds. Whole-genome sequencing was performed on one affected dog which resulted in 6,880,938 variants. As a proof of principle, we have analyzed the whole‐genome sequencing variant data of the affected dog from chr27, which includes 114,467 variants using webGQT under a recessive model. Additionally, we have used WGS data from 329 control dogs to filter out the common variants. A recessive filtering strategy is applied, allowing some heterozygote genotypes in other breeds. The affected dog is coded as “Phenotype=2” and the unaffected dogs as “Phenotype=1” in the PED file. The result of the webGQT analysis is a list of candidate variants that segregate with the disease model. webGQT detected 186 variants across different gene regions, which include six exonic variants (**Supplementary Table 2**). The six coding variants include a frameshift and stop loss novel structural variant in exon 7 of the *KRT71* gene (c.1266_1273delinsACA) which is a causative gene for curly fur. The case-study data set is available from the homepage of webGQT.

### Performance of webGQT on Phase 3 1000 Genomes Data

To evaluate the efficiency of webGQT, we compared the execution time of webGQT with *canvasDB*, an existing tool/database on 1000 Genomes data that has 4.4 billion variants from 1,092 whole genomes from 26 populations. The task is to find the population-specific variants i.e. variants present in at least 10% of the individuals in the target population and at most 1% in all the other individuals from the other populations. Previously, *canvasDB* has reported the execution times applying the same filtering strategy on the Phase 1 data set with 4.4 billion variants and 1,092 individuals (Ameur et al., 2014). Here, we have implemented webGQT with the same strategy on phase 1 data set to allow direct comparison with *canvasDB*. The filtered variants count and execution times have been reported in **Table 1**. For the majority of the populations, the execution time of webGQT on the Phase 1 data set ranged from 1 to 6 min while it took more than 30 min to find IBS, YRI, and LWK population specific variants. *canvasDB* completed the same task in the time range of 29 min to 20 hrs. On the phase 1 data set, the time required for the population-specific variant filtering by webGQT has been always several times less than *canvasDB* (**Table 1**). In addition, we have also implemented webGQT on the phase 3 data set, which has nearly twice the number of variants (8.4 billion) and more than twice the number of individuals (2,504) in phase 1 data set. Strikingly, the execution times of webGQT for population-specific variant filtering on the much larger Phase 3 data set are in the time range of 3 to 10 min, except for LWK which took 13 min to query and write the results to GUI. The maximum time taken by webGQT for both querying and writing to disk is several times less than the minimum query time taken by *canvasDB* on 78% (11/14) of the Phase 1 queries and 100% (14/14) on Phase 3 population queries, which signifies that the performance of webGQT is increased with increased cohort sizes. (**Table 1**). The conversion of VCF output to tabular format is implemented with vcfR package (Knaus and Grunwald, 2017) within webGQT.

**Table 1:** Performance comparison of webGQT with canvasDB on 1000 Genomes Phase 1 and Phase 3 data sets.

Population	Phase 1		Phase 3
	canvasDB	webGQT	webGQT
GBR	30 m	1 m 28 s	3 m 20 s
FIN	40 m	2 m 55 s	6 m 25 s
CHS	40 m	2 m 10 s	4 m 5 s
PUR	39 m	2 m 12 s	3 m 40 s
CLM	43 m	2 m 15 s	3 m 40 s
IBS	4 h 17 m	32 m 40 s	3 m 20 s
CEU	5 m	34 m 40 s	4 m 30 s
YRI	13 h 26 m	28 m 10 s	5 m 50 s
CHB	33 m	2 m 18 s	4 m 45 s
JPT	59 m	5 m 45 s	6 m 58 s
LWK	20 h 25 m	34 m 40 s	13 m 58 s
ASW	49 m	6 m 25 s	4 m 20 s
MXL	59 m	2 m 2 s	4 m 50 s
TSI	29 m	1 m 12 s	4 m 40 s

## Discussion

webGQT brings the power and performance of GQT to a wider audience, including researchers from bio/medical field and clinicians without bioinformatic expertise, by replacing the command-line interface with an intuitive web GUI. This includes pre-built modules to perform customized filtering analysis for Mendelian disease and population studies. Some previously published methods like GEMINI (Paila et al., 2013) and *canvasDB* (Ameur et al., 2014) have similar filtering modules to webGQT. However, the major distinctive feature of webGQT is its use of GQT on the backend, which uses an “sample-centric” strategy for indexing and mining large data sets and the Word-Aligned Hybrid (WAH) bitmap indices strategy for data compression that maximizes performance over all other existing methods (Layer et al., 2016). Like variant filtering tools that require pre-processing of the variant data, webGQT also requires pre-processed GQT index files which is the bottleneck for attaining better performance involving big data sets. The indexing offers webGQT an advantage in exploring massive data sets of thousands to millions of genomes while similar tools such as *canvasDB* (Ameur et al., 2014) are limited to tens of thousands of samples from whole-exome experiments, which are 1% to 2% of the genome. As shown in the results, webGQT outperformed *canvasDB* in the most complex population specific queries on the 1000 Genomes data set with twice the number of samples compared to the *canvasDB* data set.

webGQT can also be used to query non-human data sets. The case-study demonstrates the applicability of webGQT on other model species by identifying a recessively inherited known disease-causing mutation, while the existing companion method GEMINI solely supports human variation. In the case-study, the use of 329 internal controls by webGQT allowed to filter out the common variants which not only emphasizes the importance of including internal control cohorts in the absence of population frequency but also highlights the scalability of webGQT with many samples in the VCF. The scalability is further evident by implementing webGQT on 2,504 genomes from the 1000 Genomes data set. In addition, the various filtering modules across pedigree analysis and case-control studies, allows the user to apply expert knowledge to query the variant database in different possible ways. In the absence of the parent's genomes of the affected individual, case-control module facilitates to filter for specific genotypes among the affected individuals contrasting with healthy controls.

In contrast to providing the software only as a stand-alone application or only as a web application, we provide webGQT in both forms. The web application provides a resource for the 1000 Genome project Phase 3 data set *via* the web interface that allows users to query the 1000 Genomes data set by uploading the PED file defining their phenotypes of interest or population groups or by uploading custom data sets. Alternatively, the users can install their own version of webGQT as an R package on local computer (Linux/Mac OS) and can query by deploying or uploading custom GQT indexed data sets where the data will be available only to the user. webGQT can also be deployed on a local or remote server with their genomic variant data to allow multiple users within the group to query the variants by only uploading only the project specific PED file. This allows users within the group to filter variant data without installing R or the webGQT application or without requiring for independent database creation. Further, webGQT installation on local servers can be accessed or limited only to the users with the web-address to the application.

webGQT is a GQT database dependent web server and, therefore, requires pre-indexed GQT files that serve as a bottleneck to attain the best query performance among large-scale genome projects. Enabling native VCF support will require to perform the indexing of the VCF file on the server hosting the application which will consume hours of computational time especially for large-scale data sets and, therefore, disabled. webGQT is not a variant annotation tool and also does not require gene annotations in the input VCF/BCF. If an annotation exists, it is utilized only to summarize the filtered variants across the gene features. This unrequired gene annotation extends the applicability of webGQT to any model species and the user can opt to upload Annovar (Wang et al., 2010) or snpEff (Cingolani et al., 2012) pre-annotated variant data in VCF format for any species. The current version of webGQT does not natively support the popular VEP annotations, in which case the filtered variant summary across gene features are missing, although variant filtering can still be performed. Furthermore, webGQT does not support the variant data in gVCF format. While gVCF is popularly used to store variant and non-variant information from WGS samples, it is not widely supported for downstream analysis and also restricts the merging of targeted or exome samples with the genome sequencing data sets.

It is important to note that the data uploaded to our web-server is temporarily available only to the user during the user session and is not retained or distributed or stored on our servers. However, it is the user responsibility to comply with the data security and privacy while uploading sensitive or clinical human data. We encourage researchers to copy and modify the code to suit their specific research needs and local installations and also welcome the contribution of users and experts in future development of the app. The development of the app is ongoing, and we intend to improve on the speed and analysis modules.

## Conclusions

In summary, webGQT is a web application built with GQT tools with an emphasis on filtering for candidate disease-causing variants from larger cohorts of samples. We expect that webGQT suits the need of research/clinical groups that require a common platform for storing, management, and filtering variant data large-scale genomic data sets with many samples. Overall, webGQT serves as a useful tool for disease genomics e.g., for candidate disease variant filtering among families, pedigrees or larger cohorts, as well as filtering population specific variants in short time. The availability of webGQT as a web application, stand-alone installation on user computer, and a stand-alone installation on local servers of a research group allows the user to protect or share sensitive data.

## Data Availability Statement

Publicly available datasets were analyzed in this study. This data can be found here: ftp://ftp.1000genomes.ebi.ac.uk/vol1/ftp/release/20130502/, ftp://ftp.1000genomes.ebi.ac.uk/vol1/ftp/release/20110521/.

## Author Contributions

MA designed and developed the webtool, performed the analyses and drafted the paper. RL, MH, and HL contributed to model development and paper writing. All authors read and approved the final manuscript.

## Funding

This study was partially funded by Jane, Aatos Erkko and HL Foundation, the Academy of Finland (308887) and the US National Institutes of Health (RML R00HG009532).

## Conflict of Interest

RL is cofounder of Base2 Genomics LLC.

The remaining authors declare that the research was conducted in the absence of any commercial or financial relationships that could be construed as a potential conflict of interest.

## Supplementary Material

Supplementary Figure 1 **(A)** Phenotype file page of webGQT to upload the PED file with sample meta-information such as affection status, population, gender etc. The phenotype file requires IndividualID, Phenotype columns for pedigree and case-control studies. Additionally, Population and Gender columns are required for population module filters. **(B)** An example of the uploaded PED file displayed as data table after clicking “View Samples.” The summary table shows the count of number of affected, unaffected, and carrier samples in the uploaded PED file where the data table shows the phenotype file content. This page also shows the “CreateDB” button to subsequently create a phenotype sample database.

Supplementary Figure 2 **(A)** An example of variant filtering under dominant module allowing a MAF of up to 0.1 in the control cohort is shown in the figure. The progress bar during the filtering operation is shown at the bottom of the page. **(B)** An example of the Summary table displayed in the results page. Each column corresponds to different annotation databases in the input VCF/BCF file, and each row corresponds to different functional regions. The total filtered variants across each functional region are reported. **(C)** An example of the variant table returned from the filtering module is shown here. The table displays all the FLAGS present in the input VCF file in separate tab-delimited columns that can be viewed upon scrolling to the right of the table (only a few columns are shown in the figure). **(D)** An example of the barplot generated using the count of variants across different functional regions from the summary table.
