# Supplementary material for: webGQT: A Shiny Server for Genotype Query Tools for Model-Based Variant Filtering
Source: Front Genet. 2020 Mar 3;11:152. doi: 10.3389/fgene.2020.00152 (PMC7063093; doi:10.3389/fgene.2020.00152)
Supplement: Supplementary Figure 1 — (A) Phenotype file page of webGQT to upload the PED file with sample meta-information such as affection status, population, gender etc. The phenotype file requires IndividualID, Phenotype columns for pedigree and case-control studies. Additionally, Population and Gender columns are required for population module filters. (B) An example of the uploaded PED file displayed as data table after clicking “View Samples.” The summary table shows the count of number of affected, unaffected, and carrier samples in the uploaded PED file where the data table shows the phenotype file content. This page also shows the “CreateDB” button to subsequently create a phenotype sample database. [file Image_1.pdf]

(A)

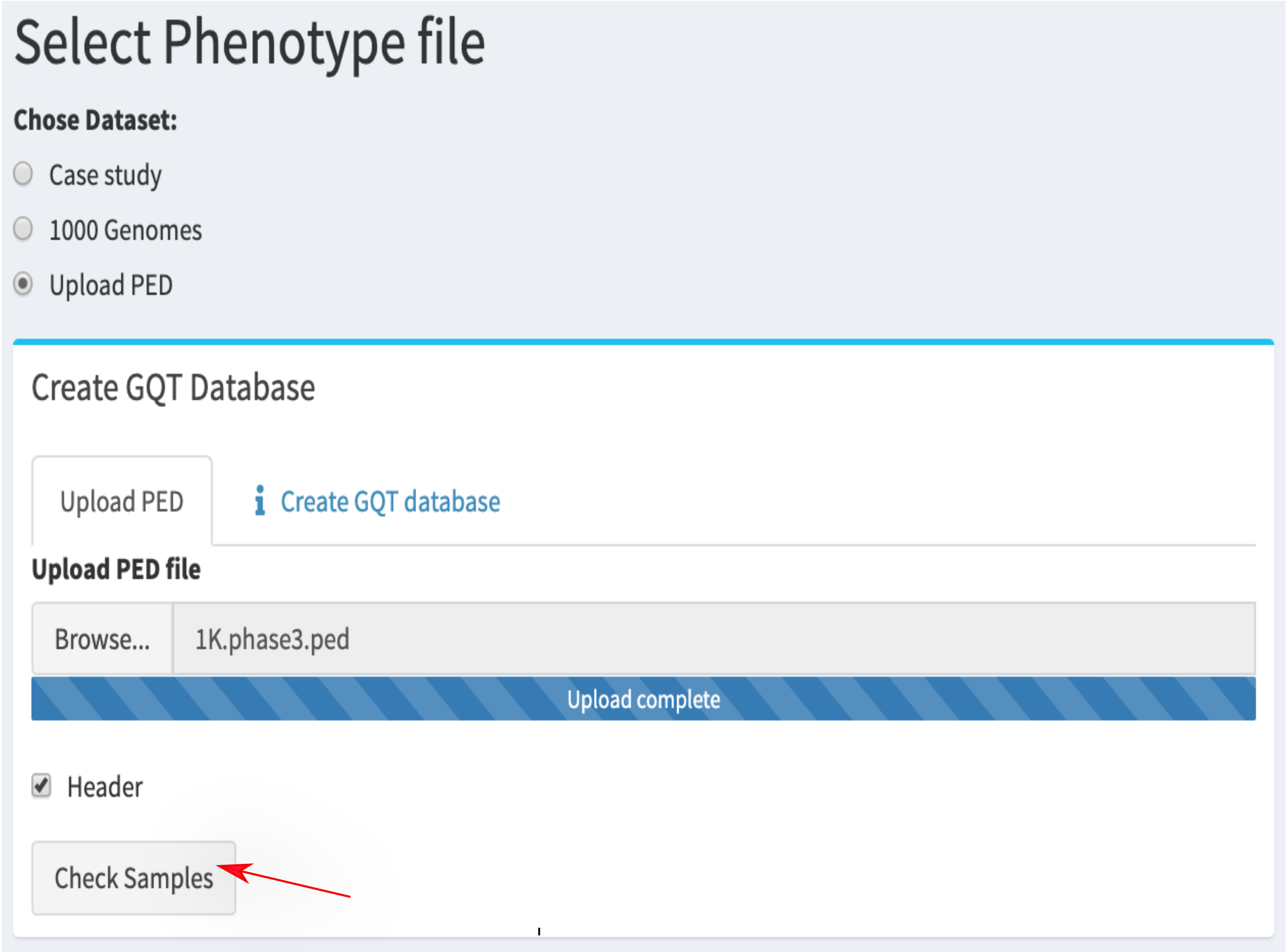
Select Phenotype file
Chose Dataset:
Case study
1000 Genomes
Upload PED
Create GQT Database
Upload PED
Create GQT database
Upload PED file
Browse...
1K.phase3.ped
Upload complete
Header
Check Samples

(B)

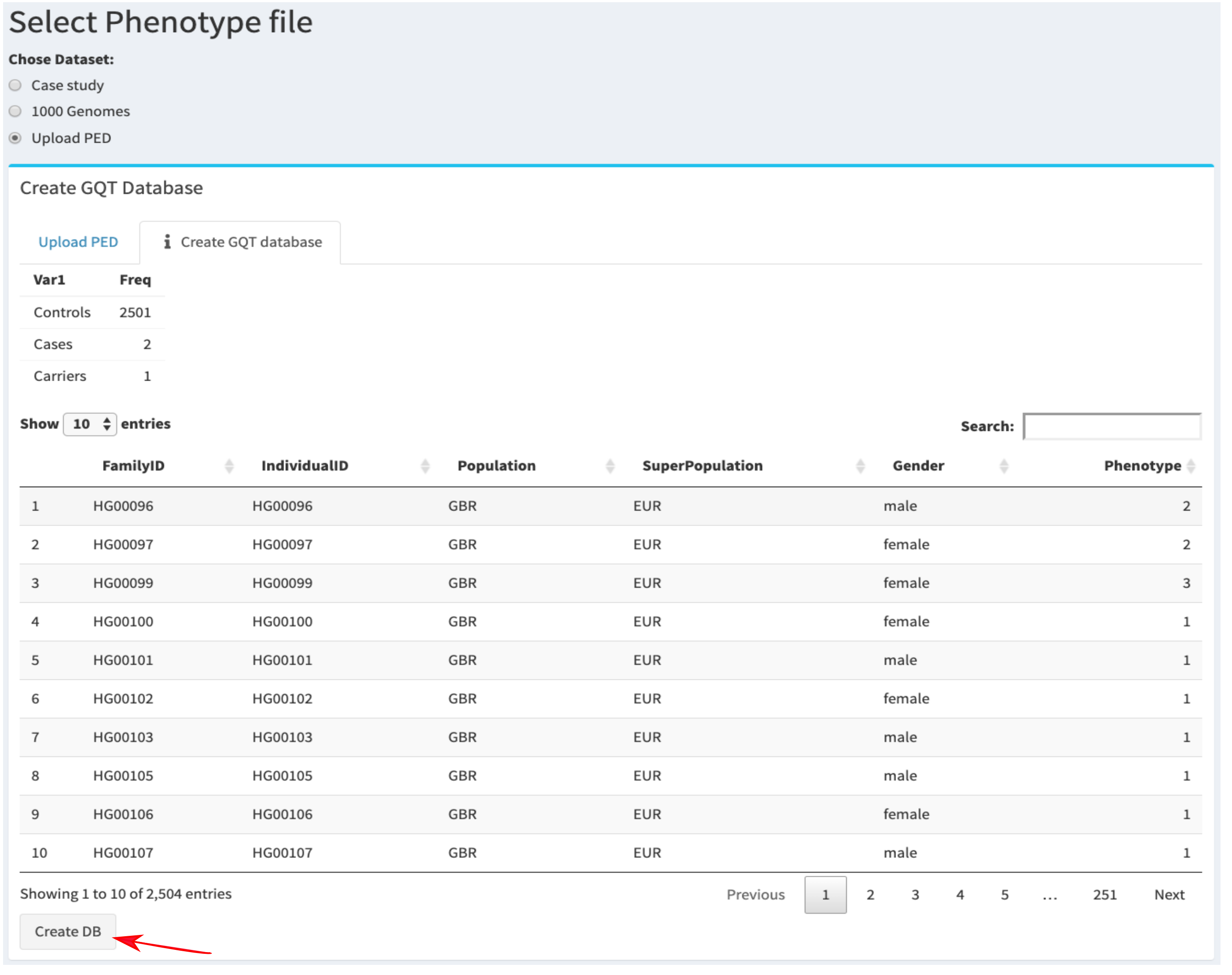
Select Phenotype file
Chose Dataset:
Case study
1000 Genomes
Upload PED
Create GQT Database
Upload PED
Create GQT database
Var1 Freq
Controls 2501
Cases 2
Carriers 1
Show 10 entries
Search:
FamilyID IndividualID Population SuperPopulation Gender Phenotype
1 HG00096 HG00096 GBR EUR male 2
2 HG00097 HG00097 GBR EUR female 2
3 HG00099 HG00099 GBR EUR female 3
4 HG00100 HG00100 GBR EUR female 1
5 HG00101 HG00101 GBR EUR male 1
6 HG00102 HG00102 GBR EUR female 1
7 HG00103 HG00103 GBR EUR male 1
8 HG00105 HG00105 GBR EUR male 1
9 HG00106 HG00106 GBR EUR female 1
10 HG00107 HG00107 GBR EUR male 1
Showing 1 to 10 of 2,504 entries
Previous 1 2 3 4 5 … 251 Next
Create DB
