# Supplementary figures and images for: webGQT: A Shiny Server for Genotype Query Tools for Model-Based Variant Filtering
Source: Front Genet. 2020 Mar 3;11:152. doi: 10.3389/fgene.2020.00152 (PMC7063093; doi:10.3389/fgene.2020.00152)

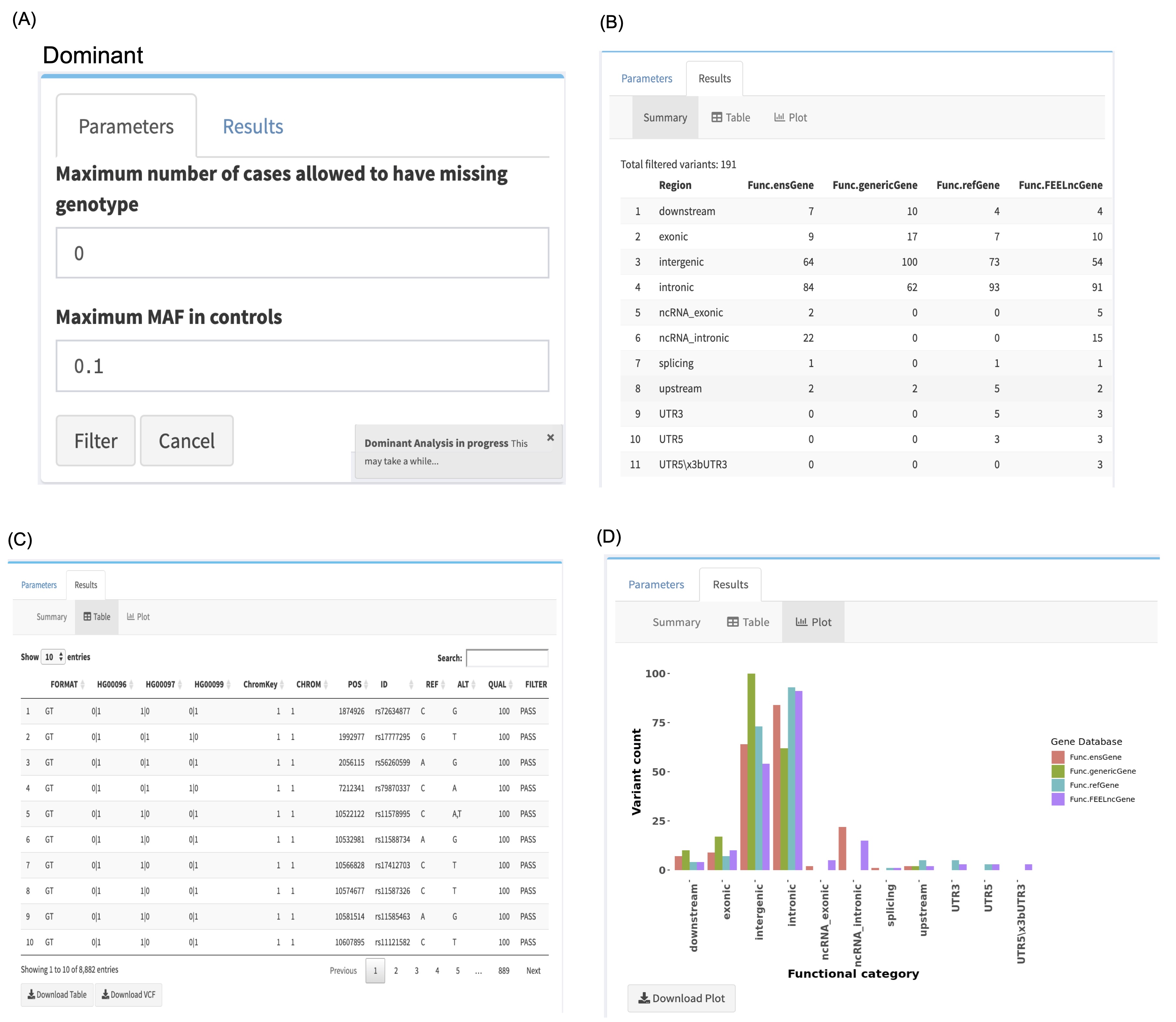

Supplement: Supplementary Figure 2 — (A) An example of variant filtering under dominant module allowing a MAF of up to 0.1 in the control cohort is shown in the figure. The progress bar during the filtering operation is shown at the bottom of the page. (B) An example of the Summary table displayed in the results page. Each column corresponds to different annotation databases in the input VCF/BCF file, and each row corresponds to different functional regions. The total filtered variants across each functional region are reported. (C) An example of the variant table returned from the filtering module is shown here. The table displays all the FLAGS present in the input VCF file in separate tab-delimited columns that can be viewed upon scrolling to the right of the table (only a few columns are shown in the figure). (D) An example of the barplot generated using the count of variants across different functional regions from the summary table. [file Image_2.jpeg]
